# A prognostic analysis of lung cancer surgery in super-elderly patients: a multicenter real-world data study

**DOI:** 10.1007/s00595-026-03431-9

**Published:** 2026-07-24

**Authors:** Makio Hayama, Mikio Okazaki, Ken Suzawa, Kazuhiko Shien, Toshiya Fujiwara, Yuji Hirami, Mototsugu Watanabe, Daisuke Okutani, Hitoshi Nishikawa, Tsuyoshi Ueno, Shinji Otani, Eisuke Matsuda, Hiroshi Yaginuma, Shinichi Toyooka

**Affiliations:** 1 Department of Thoracic Surgery, Okayama Rosai Hospital, Okayama, Japan; 2 Okayama University Thoracic Surgery Study Group, Okayama, Japan; 3 Department of General Thoracic Surgery and Breast and Endocrinological Surgery, Dentistry and Pharmaceutical Sciences, Okayama University Graduate School of Medicine, 2-5-1, Shikata-cho, kita-ku, Okayama, 700-8558 Japan

**Keywords:** Super-elderly, Lung cancer, Pulmonary resection, Overall survival, Prognostic factors, Surgical outcomes, Sarcopenia

## Abstract

**Purpose:**

With an increasing life expectancy, pulmonary resection is increasingly performed in super-elderly patients with lung cancer; however, the prognostic factors remain unclear. This study evaluated the postoperative survival and prognostic factors in patients aged ≥ 85 years.

**Methods:**

This multicenter retrospective study included 209 patients aged ≥ 85 years who underwent pulmonary resection with curative intent between 2006 and 2015. Clinical variables, including the pulmonary function, comorbidity, nutritional status, and sarcopenia, were analyzed. Survival was evaluated using the Kaplan–Meier and Cox proportional hazards models.

**Results:**

The median age was 86 years, and 119 patients (57.0%) were male. The main surgical procedures were lobectomy in 124 patients, segmentectomy in 37, and wedge resection in 47 patients. The 3-year overall survival (OS) rate was 63.6%, and the median OS was 46.5 months. A multivariate analysis identified male sex and reduced vital capacity as independent predictors of a poor survival. In clinical stage I patients, wedge resection was associated with a poorer survival than anatomical resection, although the baseline characteristics differed between the groups.

**Conclusions:**

Pulmonary resection may be feasible in carefully selected super-elderly patients. A male sex and reduced vital capacity were associated with poorer outcomes, whereas chronological age was not significantly associated with OS in this cohort.

**Supplementary Information:**

The online version contains supplementary material available at https://doi.org/10.1007/s00595-026-03431-9.

## Introduction

With continued increases in the life expectancy worldwide, the proportion of super-elderly individuals, defined as those aged ≥ 85 years, is steadily rising, particularly in high-income countries. According to the World Health Organization, the global average life expectancy at birth increased from 66.8 years in 2000 to 73.1 years in 2019 [1]. In the United States, the most recent estimates report life expectancies of 75.8 and 81.1 years for men and women, respectively [2], whereas in Western Europe, these values reach 78.9 and 84.2 years, respectively [3]. Japan continues to lead globally, with life expectancy reaching 81.09 years for men and 87.13 years for women [4].

Consequently, the number of super-elderly patients presenting with lung cancer and being considered for curative treatment has increased. National data from Japan show that, as of 2023, 17.1% of patients who underwent lung cancer surgery were aged ≥ 80 years [5]. Pulmonary resection remains the standard treatment for early stage lung cancer, and several studies have demonstrated that carefully selected octogenarians can achieve acceptable perioperative outcomes and survival after surgery [6, 7]. However, patients aged ≥ 85 years are usually included only in small numbers within such cohorts, and data specifically focused on this super-elderly population remain scarce [8].

Advancements in surgical techniques, anesthesia, perioperative management, and risk stratification have broadened the indications for pulmonary resection in older adults. Nevertheless, super-elderly patients frequently present with a reduced physiological reserve, frailty, malnutrition, and multiple comorbidities. These factors may adversely affect postoperative outcomes and long-term survival, making the balance between potential benefits and risks particularly delicate in this group [6–8]. Therefore, it is increasingly important to clarify the outcomes of lung cancer surgery in super-elderly patients and identify the prognostic factors that can help refine patient selection and perioperative strategies. This multicenter retrospective study aimed to evaluate postoperative survival and identify prognostic factors in patients aged ≥ 85 years who underwent pulmonary resection for lung cancer.

## Methods

### Study design and patients

This multicenter retrospective study was conducted by the Okayama University Thoracic Surgery Study Group (OUTSSG). Consecutive patients aged ≥ 85 years who underwent pulmonary resection with curative intent for primary lung cancer between 2006 and 2015 at 11 participating institutions were included in this study. Curative intent was determined by each participating institution. Patients who had undergone palliative surgery or documented incomplete resection, including R1 or R2 resection, were excluded from the analysis. Due to the retrospective multicenter design, the detailed surgical margin status was not centrally reviewed. This study was approved by the Institutional Review Board of Okayama University Hospital (approval No. 1910-018) and the ethics committees or institutional review boards of all other participating institutions. The requirement for written informed consent was waived because of the retrospective nature of this study. Information regarding the study was disclosed on institutional websites, and patients were given the opportunity to opt out.

### Clinical variables and assessments

Data were collected from electronic medical records and included the following variables: age, sex, smoking history, pulmonary function, nutritional and muscle status, histology, clinical and pathological staging, surgical procedure, mediastinal lymph node dissection, and postoperative complications. The Simplified Comorbidity Score (SCS), originally described by Colinet et al. [9], was used to assess comorbidities. Briefly, the SCS consists of tobacco consumption, diabetes mellitus, renal insufficiency (weighted scores: 7, 5, and 4, respectively), respiratory, neoplastic, and cardiovascular diseases, and alcoholism (weight 1 each). Patients were classified into two groups: SCS ≥ 9 and SCS < 9, according to previous studies [10, 11]. The Prognostic Nutritional Index (PNI) was calculated using the following formula: PNI = 10 × serum albumin (g/dL) + 0.005 × total lymphocyte count (/mm³), as described previously [12, 13]. In this study, a PNI value < 47 was defined as a low PNI, indicating a poor nutritional status, based on the prior literature [13]. The Psoas Muscle Index (PMI) was used to evaluate sarcopenia. The cross-sectional areas of the bilateral psoas muscles at the L3 level were measured on preoperative CT images using a Picture Archiving and Communication System (PACS) and normalized for height squared (cm²/m²). PMI was calculated as follows: (bilateral psoas area at L3) / height². The cutoff values for sarcopenia were defined as 6.36 cm²/m² for men and 3.92 cm²/m² for women, according to Hamaguchi et al. [14]. The preoperative pulmonary function was evaluated using vital capacity (VC) and forced expiratory volume in one second (FEV₁.₀%). VC < 80% predicted was defined as reduced vital capacity, and FEV₁.₀% <70% was defined as a FEV₁.₀%.

### Surgical procedures and postoperative evaluation

The surgical approaches included open thoracotomy and video-assisted thoracic surgery (VATS). Limited resection was defined as wedge resection only, whereas segmentectomy and lobectomy were categorized as anatomical resections. Because of the retrospective multicenter design, no uniform predefined criteria were applied for selecting wedge resection versus anatomical resection. The choice of surgical procedure was determined by the treating surgeons at each institution based on a comprehensive assessment of tumor-related factors, such as tumor size, location, and clinical stage, as well as patient-related factors, including pulmonary function, comorbidities, and overall surgical risk. Wedge resection is often selected as a compromised procedure in patients considered unable to tolerate anatomical resection because of limited pulmonary reserve or substantial comorbidity burden. The SCS was calculated retrospectively for the present analysis and it was not used as a formal criterion for procedure selection during the study period. In this study, mediastinal lymph node dissection was not routinely performed in patients who underwent wedge resection. Postoperative complications were defined as events occurring within 30 days after surgery or during the same hospitalization period. All complications were evaluated as binary variables (presence vs. absence) for survival analysis. The causes of death were determined based on the medical records of each participating institution. Cancer-specific survival was defined as the interval from surgery to death due to lung cancer. Deaths attributed to lung cancer were treated as events, whereas deaths from other causes and deaths of unknown causes were censored at the date of death.

### Statistical analysis

OS was defined as the time from surgery to death from any cause or the last follow-up. Survival curves were generated using the Kaplan–Meier method, and differences were evaluated using the log-rank test. For variables with missing data, percentages and analyses were calculated using the number of patients with available data for each variable as denominators. Missing data were not imputed. Univariate analyses were performed using the available cases for each variable. For the multivariable Cox proportional hazards model, patients were included if data were available for all variables included in the specific model. A primary multivariable Cox proportional hazards model was constructed to identify preoperative patient-related prognostic factors that could be used for surgical decision-making. Therefore, preoperative patient-related variables with a p-value < 0.20 in the univariate analysis were included in the model. Postoperative complications and the pathological stage were not included because they were not available at the time of preoperative decision-making, and surgical procedure was not included in the primary model because procedure selection was strongly influenced by the patient’s background and it was evaluated separately. Comparisons of the clinicopathological backgrounds among the groups were performed using the χ² test or Fisher’s exact test for categorical variables, and continuous variables were compared using Student’s *t-*test or Mann–Whitney U test, as appropriate. All statistical analyses were performed using the freely available software program EZR (a graphical interface for R) [15].

## Results

### Patient characteristics

A total of 209 patients aged ≥ 85 years who underwent curative-intent pulmonary resection for primary lung cancer were included in this study. The median age was 86 years (interquartile range 85–89), and 119 patients (57.0%) were male (Table 1). The mean PNI was 49.51 ± 8.52. The mean PMI was 5.10 ± 1.30 cm²/m² for men and 4.30 ± 1.39 cm²/m² for women. Sarcopenia, defined according to the criteria of Hamaguchi et al. [14], was observed in 92 patients (63.9% of the total). The mean SCS was 8.4 ± 2.3, and 31.3% of the patients had SCS ≥ 9. The clinicopathological characteristics of the patients are summarized in Table 1.

**Table 1:** Clinicopathological characteristics of super-elderly patients who underwent pulmonary resection for lung cancer

Variables	
Median Age (IQR, years) (*n*=209)	86 (85-89)
Sex (Male/Female)	119 (57.0%) / 90 (43.0%)
Smoking history (Present/Absent)	115 (55.8%) / 91 (44.2%)
%VC <80% (Yes/No)	33 (16.9%) / 162 (83.1%)
FEV1.0% <70% (Yes/No)	72 (39.1%) / 112 (60.9%)
PNI (*n*=171)	49.51 ± 8.52
PMI (cm²/m², Male *n*=79 /Female *n*=65)	5.10 ± 1.30 / 4.30 ± 1.39
Sarcopenia (Present / Absent)	92 (63.9%) / 52 (36.1%)
SCS (≥9 / <9)	65 (31.3%) / 143 (68.7%)
Histology (Adenocarcinoma / Sq / Others)	138 (66.0%) / 60 (28.7%) / 11 (5.3%)
Clinical stage (I / II–IV)	167 (80.0%) / 42 (20.0%)
Surgical approach (VATS / Open)	166 (85.6%) / 28 (14.4%)
Surgical Procedure (Lobectomy / Segmentectomy / wedge resection)	124 (59.6%)/37 (17.8%)/ 47 (22.6%)
Mediastinal LN dissection (Yes / No)	112 (58.0%) / 81 (42.0%)
Pathological stage (I / II–IV)	158 (75.6%) / 51 (24.4%)
Postoperative complications (Yes / No)	53 (25.4%) / 156 (74.6%)

Values are presented as mean ± standard deviation or number of patients (%)For variables with missing data, percentages and summary statistics were calculated using the number of patients with available data as the denominatorIQR: Interquartile Range; PMI: Psoas Muscle Index; PNI: Prognostic Nutritional Index; SCS: Simplified Comorbidity Score; Sq: Squamous cell carcinoma; VATS: Video Assisted Thoracic Surgery

### Postoperative complications

Postoperative complications occurred in 53 patients (25.4%), with 65 complications recorded. Complications occurred in 38 of 124 patients (30.6%) who underwent lobectomy, 7 of 37 patients (18.9%) who underwent segmentectomy, and 7 of 47 patients (14.9%) who underwent wedge resection. The remaining patient underwent wedge resection combined with chest wall resection and developed a postoperative complication. The most frequent complications were prolonged air leakage (*n* = 16), arrhythmia (*n* = 10), pneumonia (*n* = 6), and post-operative delirium (*n* = 5). No in-hospital or 30-day postoperative deaths were observed.

### Survival outcomes and prognostic factors

The median observation period was 38.2 months (range, 1–123). During this period, 111 patients died, including 40 cancer-related deaths, 15 pneumonia-related deaths, 7 cardiovascular deaths, 5 cerebrovascular deaths, 2 interstitial pneumonia deaths, 2 respiratory failure deaths, 4 deaths from other malignancies, 8 deaths from miscellaneous causes, and 28 deaths of unknown cause.

**Fig. 1:**
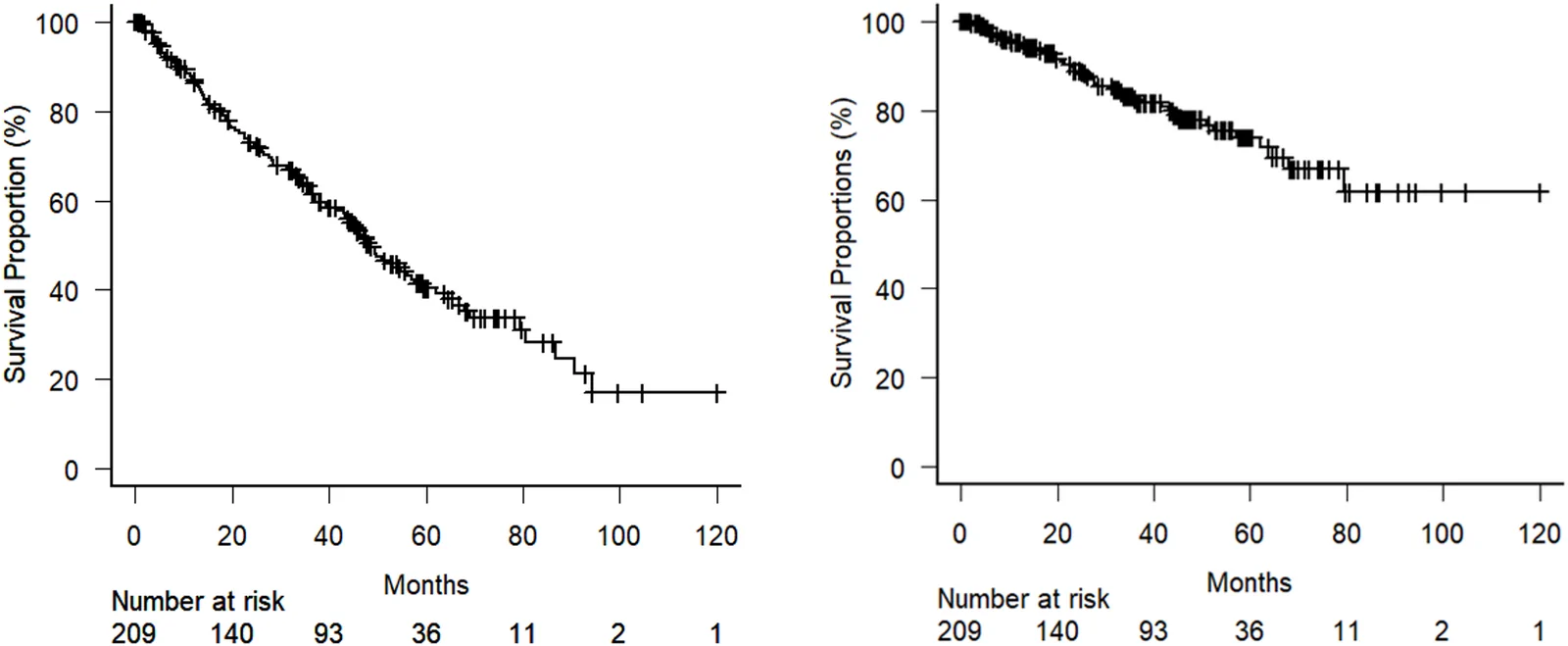
(**A**) Kaplan–Meier curve for overall survival in all 209 super-elderly patients. (**B**) Kaplan–Meier curve for cancer-specific survival in all 209 super-elderly patients

**Fig. 2:**
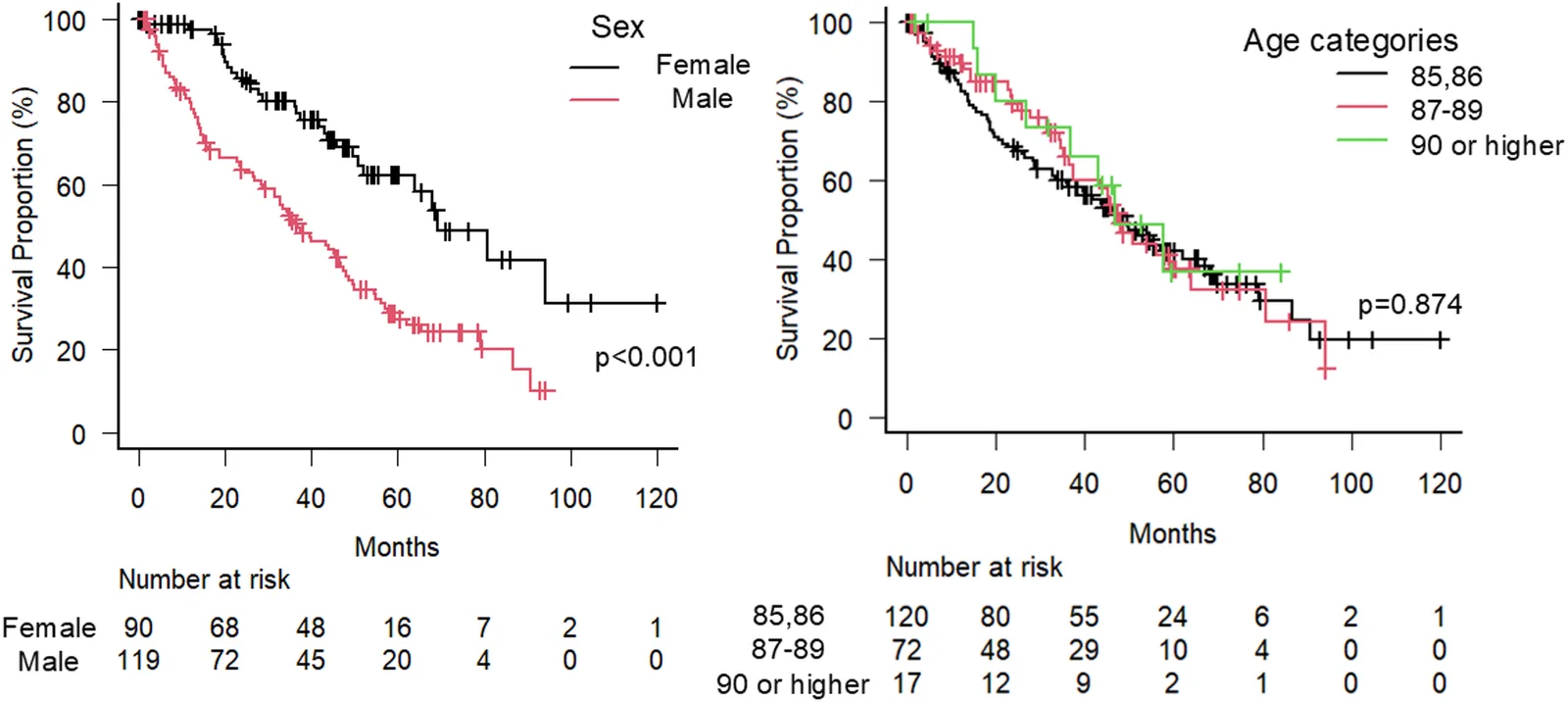
(**A**) Kaplan–Meier curves for overall survival according to sex. (**B**) Kaplan–Meier curves for overall survival among three age categories

The median OS for all patients was 46.5 months, and the 3-year OS rate was 63.6% (Fig. 1A). The 3-year cancer-specific survival rate was 83.6% (Fig. 1B). A univariate analysis of clinicopathological factors and OS is shown in Table 2. Male sex, smoking history, reduced VC, high SCS (≥ 9), sarcopenia, squamous histology, pathological stage II–IV, and the presence of postoperative complications were significantly associated with poor survival. Kaplan–Meier curves demonstrated that survival was significantly poorer in male than in female patients (Fig. 2A). A multivariable Cox regression analysis for preoperative variables was also performed and demonstrated that male sex and reduced vital capacity were independent predictors of a poor prognosis (Table 3).

**Table 2:** Univariate analysis of prognostic factors for overall survival

Variables				Median OS (months)	95% CI (months)	3-year OS rate (%)	*p*-value
Sex	Male (*n*=119)			36.6	28.5–46.7	50.4	< 0.001
	Female (*n*=90)			69.1	52.2–NA	80.3	
Age	85,86 (*n*=120)			49.5	35.9-62.2	59.2	0.686
	≥87 (*n*=89)			47.9	37.3-63.4	67.5	
Smoking history	Absent (*n*=91)			69.1	54.4–NA	81.3	< 0.001
	Present (*n*=115)			35.2	26.6–45.6	48.6	
FEV1.0%	≥70% (*n*=112)			47.9	39.5–63.9	62.8	0.553
	<70% (*n*=72)			46.5	36.6–54.7	63.6	
%VC	≥80% (*n*=162)			54.5	45.9–68.0	67.5	0.022
	<80% (*n*=33)			35.9	19.6–46.7	46.9	
SCS	≥9 (*n*=65)			32.7	18.8–43.0	43.9	< 0.001
	<9 (*n*=143)			63.9	49.8–69.0	71.6	
Sarcopenia	Present (*n*=92)			46.5	34.2–54.5	60.2	0.018
	Absent (*n*=52)			63.9	54.7–NA	77.6	
PNI	≥47 (*n*=101)			57.7	45.9–68.0	71.6	0.321
	<47 (*n*=70)			43.0	31.5–86.8	55.8	
Histology	Adenocarcinoma (*n*=138)			57.7	47.5–69.1	73.6	< 0.001
	Sq (*n*=60)			26.6	26.6–34.5	34.2	
	Others (*n*=11)			NA	NA–27.8	77.9	
Clinical stage	I (*n*=167)			49.8	40.0–65.5	63.9	0.218
	II–IV (*n*=42)			44.0	25.9–62.2	58.1	
Surgical approach	Open (*n*=28)			41.6	18.7–59.2	54.6	0.147
	VATS (*n*=166)			49.5	43.4–63.9	64.5	
Surgical Procedure		Wedge resection (*n*=47)		34.5	15.8–65.5	49.5	0.093
		Segmentectomy or Lobectomy (*n*=161)		49.6	45.2–59.2	66.4	
Mediastinal lymph node resection			Yes (*n*=112)	54.5	46.7–63.9	66.7	0.073
			No (*n*=81)	41.6	31.3–54.7	57.0	
Pathological stage			I (*n*=158)	54.7	45.9–79.3	68.3	< 0.001
			II–IV (*n*=51)	31.5	20.6–49.5	46.8	
Postoperative complications			Yes (*n*=53)	41.6	21.6–48.7	55.1	0.007
			No (*n*=156)	54.7	43.4–69.1	65.2	

The total cohort included 209 patients. The number of missing values for each variable can be calculated as 209 minus the number analyzed. Missing data were not imputedCI: Confidence interval; NA: Not Available; OS: Overall survival; PNI: Prognostic Nutritional Index; SCS: Simplified Comorbidity Score; Sq: Squamous cell carcinoma

**Table 3:** Multivariable analysis of preoperative prognostic factors for overall survival

Variable	HR (95% CI)	*p*-value
Male sex	2.704 (1.554–4.704)	< 0.001
Smoking history (Present vs. Absent)	1.142 (0.4379–2.978)	0.783
**VC <80%**	**2.013 (1.082–3.745)**	**0.027**
Sarcopenia (Present vs. Absent)	1.458 (0.838–2.540)	0.185
SCS ≥9	1.505 (0.884–2.566)	0.132
Histology (Ad vs. non-Ad)	0.909 (0.558–1.479)	0.674

The multivariable Cox proportional hazards model included 142 patients with available data for all variables included in the model. Ad: Adenocarcinoma; CI: Confidence interval; HR: Hazard ratio; SCS: Simplified Comorbidity Score; VC: Vital capacity

### Comparison of prognosis among three age categories in this cohort

In the univariate analysis comparing patients aged 85–86 years and those aged ≥ 87 years, there was no significant difference in OS. The age distribution (Supplemental Fig. 1) clearly demonstrated that most patients in this cohort were aged 85 or 86 years, whereas patients aged ≥ 90 years accounted for a smaller proportion. Based on this distribution, the cohort was divided into three age categories (85–86, 87–89, and ≥ 90 years) for subsequent analyses. There were no significant differences in the clinicopathological backgrounds among these three groups (Supplemental Table 1), and no significant differences in OS were observed among the three age groups (Fig. 2B). Consistently, age did not emerge as a significant predictor in the univariate analyses and was therefore not included in the multivariable model.

### Comparison between wedge resection and anatomical resection in clinical stage I patients

**Fig. 3:**
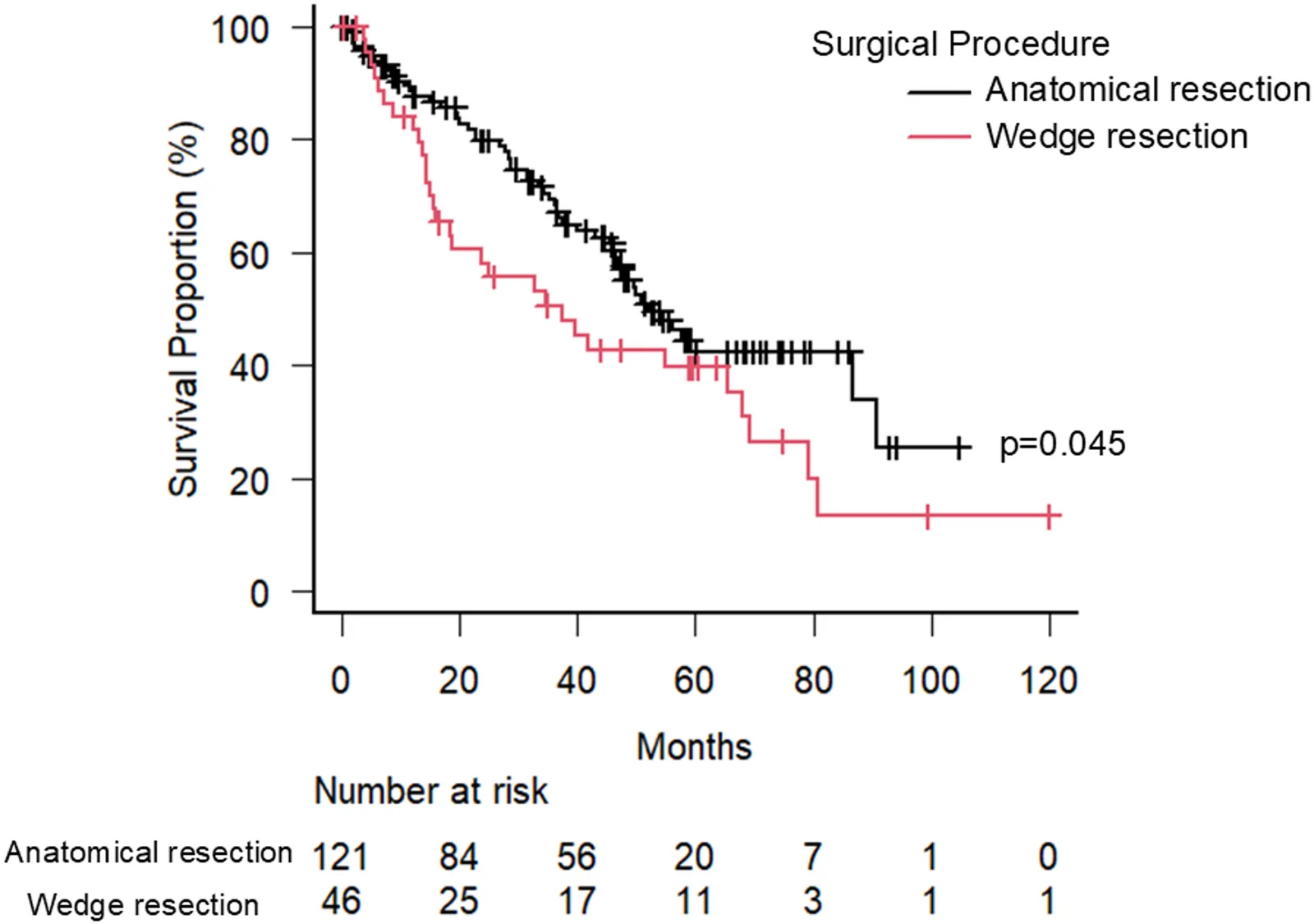
Kaplan–Meier survival curves for patients with clinical stage I disease undergoing wedge resection or anatomical resection

In this study, 46 of 47 patients (97.9%) in the wedge resection group were in clinical stage I, whereas 40 of 161 patients (24.8%) in the anatomical resection group were in stage II or higher. Therefore, the prognostic analysis between wedge resection and anatomical resection was limited to patients with clinical stage I disease (Table 4). A Kaplan–Meier analysis showed that the wedge resection group had a significantly poorer OS than the anatomical resection group (*p* = 0.045; Fig. 3).

**Table 4:** Comparison between wedge resection and anatomical resection (segmentectomy or lobectomy) in clinicopathological characteristics of the clinical stage I patients

Variables	Wedge Resection	Anatomical Resection	*p*-value
Total patients	46	121	
Median age (IQR, years)	86 (85-88) (*n*=46)	86 (85-88) (*n*=121)	0.677
Sex (Male/Female)	31/15	61/60	0.057
Smoking history (Present/Absent)	32/13	59/61	0.014
%VC <80%(Yes / No)	10/30	16/98	0.141
FEV1.0%<70% (Yes / No)	21/17	37/72	0.033
PNI (≥47 / <47)	23/15	61/39	1
Sarcopenia (Present / Absent)	26/5	47/37	0.008
SCS (≥9 / <9)	21/25	28/92	0.007
Median total tumor size (IQR, mm)	21 (15-28) (*n*=45)	31 (22-35) (*n*=118)	< 0.001
Histology (Adenocarcinoma / Sq / Others)	25/20/1	87/28/6	0.032
Surgical approach (VATS / Open)	40/2	100/13	0.358
Mediastinal LN dissection (Yes / No)	0/46	79/28	< 0.001
Pathological stage (I / II–IV)	44/2	100/21	0.042
Postoperative complications (Yes / No)	6/40	31/90	0.067

IQR: Interquartile Range; PNI: Prognostic Nutritional Index; SCS: Simplified Comorbidity Score; Sq: Squamous cell carcinoma; VATS: Video Assisted Thoracic Surgery

## Discussion

This multicenter retrospective study investigated the prognostic factors in patients aged ≥ 85 years who underwent pulmonary resection for primary lung cancer. The results demonstrated that male sex and reduced vital capacity were independent preoperative predictors of poor survival. Despite the advanced age of this cohort, the perioperative complication rate and intermediate-term survival outcomes suggest that surgical treatment remains a feasible and acceptable option for carefully selected patients.

Our findings showed that male patients had significantly poorer survival outcomes, consistent with previous studies suggesting sex-related biological differences in lung cancer behavior [16, 17]. Further examination of sex-based differences in our cohort revealed that male patients presented with several unfavorable baseline characteristics that may have contributed to their worse prognoses. As shown in Supplemental Table 2, men were significantly more likely to have a history of smoking and reduced FEV₁.₀%, sarcopenia, and higher SCS. They also had a higher prevalence of squamous cell carcinoma, which is known to be associated with a poorer survival than adenocarcinoma [18–20]. These differences suggest that the poorer survival in male patients may be due not only to biological sex differences but also to a higher burden of adverse clinical and pathological factors. A reduced vital capacity emerged as another significant predictor of a poor prognosis, highlighting the close relationship between preoperative respiratory reserve and long-term outcomes in elderly patients [21]. It is reasonable to assume that a reduced preoperative pulmonary function results in an even lower postoperative respiratory capacity, which may further compromise the survival outcomes. The ECOG performance status and formal frailty assessments were not uniformly collected in this retrospective cohort study. Instead, sarcopenia based on the psoas muscle index at the L3 level was evaluated in patients with available CT images as an objective marker of a reduced physical reserve. Sarcopenia was associated with poorer OS in the univariate analysis, but it was not retained as an independent prognostic factor in the multivariate analysis. This may partly reflect the high prevalence of sarcopenia in this super-elderly cohort and its overlap with sex and body composition. In particular, many patients aged ≥ 85 years, especially men, may meet the current CT-based criteria for sarcopenia, which may limit its discriminatory ability in this age group. Therefore, sarcopenia should be interpreted as one component of a comprehensive preoperative assessment rather than as a sole determinant of the surgical indications.

Postoperative complications occurred in 25.4% of the patients. This complication rate is comparable to those reported previously in octogenarians and supports the notion that pulmonary resection can be safely performed in selected super-elderly patients [6–8, 21–23]. However, complications such as pneumonia and prolonged air leakage not only extend hospitalization, but may also delay rehabilitation and functional recovery, thereby negatively impacting long-term outcomes. As expected, a more advanced pathological stage was also associated with a worse survival, reflecting the intrinsic prognostic weight of the tumor burden rather than age-specific factors.

When only clinical stage I cases were compared, the wedge resection group showed poorer OS than the anatomical resection group. However, this finding should be interpreted cautiously because the choice of procedure was not randomized and it was strongly influenced by the patient’s background. Wedge resection was often selected for patients with limited pulmonary reserve or substantial comorbidity burden, and the wedge resection group had less favorable baseline characteristics, including poorer pulmonary function, more frequent sarcopenia, higher SCS, and less frequent mediastinal lymph node dissection than the lobectomy group. In contrast, the tumor size was smaller in the wedge resection group than in the anatomical resection group. Therefore, the observed survival difference may reflect confounding by the indication rather than the oncological inferiority of a wedge resection.

For patients who can tolerate only a limited resection because of severely impaired respiratory function or substantial comorbidities, non-surgical treatment strategies, such as stereotactic body radiotherapy (SBRT), may also be considered as part of individualized treatment decision-making. Pooled randomized data have suggested comparable survival between SBRT and surgery in operable stage I disease [24], whereas large population-based analyses in elderly patients have generally demonstrated superior long-term survival with surgical resection [25]. However, patients aged ≥ 85 years have rarely been examined separately from other age groups. Because our study included only surgically treated patients, a direct comparison with SBRT was not feasible, and further comparative research on the super-elderly population is warranted.

Although the super-elderly population represents a highly selected surgical cohort, the observed perioperative results and survival indicate that pulmonary resection is feasible in this age group. Preservation of postoperative functional independence is particularly important when considering surgery in patients aged ≥ 85 years. In this study, the 3-year OS rate was 63.6%, which appears favorable when viewed in the context of the average life expectancy at this age [4]. These findings support the idea that when comorbidities and physiological reserves are thoroughly evaluated, surgical treatment can be justified even in very advanced age. Furthermore, as older age is generally associated with a worse prognosis, we examined the outcomes across three age categories, including patients aged ≥ 90 years, to assess whether further aging within this super-elderly cohort influenced survival. In the present cohort, no significant differences in OS were observed among the three age groups. However, this finding should be interpreted cautiously because only 17 patients were aged ≥ 90 years. The absence of a statistically significant difference does not indicate equivalence between the age groups. These results suggest that chronological age alone may not be sufficient to determine surgical eligibility in carefully selected super-elderly patients. Further studies with larger numbers of nonagenarians are needed to clarify the safety and appropriateness of pulmonary resection in this age group.

This study has several limitations. First, the CSS results should be interpreted with caution. During follow-up, approximately one-fourth of the deaths were classified as unknown causes. In this super-elderly population, some patients died in nursing facilities or other non-hospital settings, and detailed clinical information immediately before death was not always available. Therefore, deaths that could not be confidently attributed to lung cancer were classified as unknown and censored in the CSS analysis. This approach may have resulted in an overestimation of CSS if some deaths of unknown cause were actually related to lung cancer. Additionally, a competing risk analysis was not performed, which is another limitation given the high likelihood of non-cancer mortality in this population. Accordingly, OS may be a more robust endpoint than CSS in this cohort of patients.

Second, the retrospective, multicenter design is inherently subject to some selection bias. Patient management strategies, surgical indications, and procedure selection may have varied among the participating institutions. In particular, the comparison between wedge resection and anatomical resection is susceptible to confounding by the indications, and detailed information regarding the consolidation-to-tumor ratio or whether limited resection was intentional or compromised was not available in this study.

Third, the patients in this study underwent surgery between 2006 and 2015, which should be considered when interpreting the present findings. Since then, perioperative management, minimally invasive surgical approaches, sublobar resection strategies, and geriatric assessment have substantially advanced, potentially expanding surgical indications and treatment options for older patients compared with the study period. In addition, nonsurgical treatments, including SBRT, have evolved. Therefore, although the present study provides meaningful evidence based on real-world clinical practice during that period, changes in the therapeutic landscape should be considered when applying these findings to contemporary practice.

Fourth, the ECOG performance status, comprehensive geriatric assessment, formal frailty indices, postoperative QoL, and preservation of functional independence were not uniformly evaluated. Although sarcopenia was assessed using preoperative CT images, it could not be evaluated in all patients and may have limited discriminatory ability in this age group because of its high prevalence in individuals aged ≥ 85 years. In addition, postoperative complications were analyzed primarily as binary variables, and severity grading using the Clavien–Dindo classification was not available. Furthermore, the multivariable Cox model included 142 patients with available data for all variables included in the model, and the possibility of bias due to missing data cannot be excluded from this study.

Finally, this study included only Japanese patients treated at specialized centers, which may limit the generalizability of our findings to other ethnic groups and healthcare systems.

Nevertheless, this multicenter study included a relatively large number of patients aged ≥ 85 years and provides valuable insights into the feasibility and prognostic factors of lung cancer surgery in this super-elderly population.

## Conclusion

This multicenter analysis showed that a male sex and reduced vital capacity were independent preoperative predictors of a poor survival in carefully selected patients aged ≥ 85 years who underwent pulmonary resection for lung cancer. Chronological age within this cohort was not significantly associated with OS, but this finding should be interpreted cautiously because few patients were aged ≥ 90 years. Pulmonary resection may remain a feasible treatment option when appropriate patient selection, individualized procedure selection, and meticulous perioperative management are ensured.

## Supplementary Information

Below is the link to the electronic supplementary material.

Supplementary Material 1

Supplementary Figure 1. Age distribution of patients Vertical dashed lines indicate the boundaries between the three age categories (85–86, 87–89, and ≥90 years).

## References

[1] World Health Organization. Life expectancy and healthy life expectancy. 2020. https://www.who.int/data/gho. Accessed 12 Oct 2025.

[2] Centers for Disease Control and Prevention, US life expectancy. 2023. NCHS Data Brief No. 521. 2024. https://www.cdc.gov/nchs/products/databriefs/db521.htm. Accessed 12 Oct 2025.

[3] Institut national d’études démographiques. Life expectancy in developed countries. https://www.ined.fr/en. Accessed 12 Oct 2025.

[4] Ministry of Health, Labour and, Welfare J. Summary of the simplified life tables, 2024. 2025. https://www.mhlw.go.jp/toukei/saikin/hw/life/life24/index.html. Accessed 12 Oct 2025.

[5] Yoshimura N, Sato Y, Endo S, Nakanishi K, Shiose A, Okada M, et al. Thoracic and cardiovascular surgeries in Japan during 2023. Gen Thorac Cardiovasc Surg. 2025;73:526–65. 10.1007/s11748-025-02128-z PMC12174170 40377897

[6] Miura N, Kohno M, Ito K, Suzuki K, Taniguchi Y, Saito H, et al. Lung cancer surgery in patients aged 80 years or older: an analysis of risk factors, morbidity, and mortality. Gen Thorac Cardiovasc Surg. 2015;63:401–5. 10.1007/s11748-015-0546-7 25868520

[7] Vazirani J, Moraes J, Barnett S, Wigfield C, Naidu B, Kalkat M, et al. Outcomes following resection of non small cell lung cancer in octogenarians. ANZ J Surg. 2018;88:1322–7. 10.1111/ans.14861 30277303

[8] Ichinokawa H, Takamochi K, Fukui M, Matsunaga T, Suzuki K, Oh S, et al. Surgical results and prognosis of lung cancer in elderly Japanese patients aged over 85 years: comparison with patients aged 80–84 years. Gen Thorac Cardiovasc Surg. 2021;69:67–75. 10.1007/s11748-020-01426-y 32627148

[9] Colinet B, Jacot W, Bertrand D, Lacombe S, Bozonnat MC, Daurès JP, et al. A new simplified comorbidity score as a prognostic factor in non-small-cell lung cancer patients: description and comparison with the Charlson’s index. Br J Cancer. 2005;93:1098–105. 10.1038/sj.bjc.6602836 PMC2361505 16234816

[10] Leung B, Shokoohi A, Al Hashami Z, Miao Q, Joy AA, Gyawali B, et al. Improved uptake and survival with systemic treatments for metastatic non small cell lung cancer: younger versus older adults. BMC Cancer. 2023;23:360. 10.1186/s12885-023-10800-x PMC10114445 37076834

[11] Kang EJ, Choi YJ, Lee SR, Kim JY, Kim YJ, Lee SY, et al. Impact of subsequent chemotherapy on the survival of elderly patients with extensive stage small cell lung cancer. Korean J Intern Med. 2020;35:1468–76. 10.3904/kjim.2019.136 PMC7652651 32336056

[12] Okada S, Shimada J, Kato D, Tsunezuka H, Teramukai S, Inoue M, et al. Clinical significance of prognostic nutritional index after surgical treatment in lung cancer. Ann Thorac Surg. 2017;104:296–302. 10.1016/j.athoracsur.2017.01.085 28433217

[13] Takahashi Y, Horio H, Hato T, Harada M, Matsutani N, Kawamura M, et al. Prognostic impact of the prognostic nutritional index after surgery for lung cancer. Ann Thorac Surg. 2019;108:908–14.

[14] Hamaguchi Y, Kaido T, Okumura S, Kobayashi A, Hammad A, Tamai Y, et al. Proposal for new diagnostic criteria for sarcopenia based on psoas muscle mass at the L3 level. Am J Clin Nutr. 2016;103:979–85. 10.1016/j.nut.2016.04.003 27292773

[15] Kanda Y. Investigation of the freely available easy-to-use software ‘EZR’ for medical statistics. Bone Marrow Transpl. 2013;48:452–8. 10.1038/bmt.2012.244 PMC3590441 23208313

[16] Hishida T, Yoshida J, Nishimura M, Nishiwaki Y, Nagai K, Ohe Y, et al. Gender differences in long-term survival after resection of non-small cell lung cancer: a multicenter analysis. J Thorac Cardiovasc Surg. 2013;145:676–82.

[17] Yano T, Miura N, Takenaka T, Haro A, Okamoto T, Morodomi Y, et al. Prognostic difference between male and female patients with resected non-small cell lung cancer. Lung Cancer. 2019;134:91–6.

[18] Wang BY, Huang JY, Chen HC, Lin CH, Lin SH, Hung WH, et al. The comparison between adenocarcinoma and squamous cell carcinoma in lung cancer patients. J Cancer Res Clin Oncol. 2020;146:43–52. 10.1007/s00432-019-03079-8 PMC11804334 31705294

[19] Izaki Y, Mimae T, Kagimoto A, Nakahara R, Yonehara S, Miyata Y, et al. Differences in postoperative prognosis between early stage lung adenocarcinoma and squamous cell carcinoma. Jpn J Clin Oncol. 2024;54:813–21. 10.1093/jjco/hyae049 38677985

[20] Hao B, Li X, Wan S, Wang J, Chen H, Zhou Z, et al. Squamous cell carcinoma predicts worse prognosis than lung adenocarcinoma in IA stage non small cell lung cancer patients: a population based propensity score matching analysis. Front Surg. 2022;9:944032. 10.3389/fsurg.2022.944032 PMC9461700 36090323

[21] Park B, Lee G, Kim HK, Choi YS, Zo JI, Shim YM, et al. A retrospective comparative analysis of elderly and younger patients undergoing pulmonary resection for stage I non-small cell lung cancer. World J Surg Oncol. 2016;14:13. 10.1186/s12957-015-0762-8 PMC4717591 26787343

[22] Saji H, Tsuboi M, Yoshida K, Kato Y, Nomura M, Matsumoto I, et al. Prognostic impact of postoperative complications after curative resection for non-small cell lung cancer. Ann Thorac Surg. 2017;103:287–94.

[23] Maeda R, Yoshida J, Ishii G, Hishida T, Nishimura M, Nagai K, et al. Impact of postoperative morbidity on long-term survival after resection for non-small cell lung cancer. J Thorac Oncol. 2015;10:1218–24.

[24] Chang JY, Senan S, Paul MA, Mehran RJ, Louie AV, Balter P, et al. Stereotactic ablative radiotherapy versus lobectomy for operable stage I non-small-cell lung cancer: a pooled analysis of two randomised trials. Lancet Oncol. 2015;16:630–7. 10.1016/S1470-2045(15)70168-3 PMC4489408 25981812

[25] Shirvani SM, Jiang J, Chang JY, Welsh JW, Likhacheva A, Buchholz TA, et al. Lobectomy, sublobar resection, and stereotactic ablative radiotherapy for early-stage non-small cell lung cancers in the elderly. JAMA Surg. 2014;149:1244–53. 10.1001/jamasurg.2014.556 PMC4401470 25321323

