# Supplementary material for: A prognostic analysis of lung cancer surgery in super-elderly patients: a multicenter real-world data study
Source: Surg Today. 2026 Jul 24;56(11):2867–75. doi: 10.1007/s00595-026-03431-9 (PMC13722745; doi:10.1007/s00595-026-03431-9)
Supplement: Supplementary file 1 [file 595_2026_3431_MOESM1_ESM.docx]

**Supplemental Table 1. Comparison among age categories in clinicopathological characteristics of the patients.**

| **Variable** | **Age category 1**  **(85, 86)** | **Age category 2**  **(87-89)** | **Age category 3**  **(90 or higher)** | **p-value** |
| --- | --- | --- | --- | --- |
| Number of patients | 120 | 72 | 17 |  |
| Age median (IQR, years) | 85 (85-86) | 88 (87-89) | 91 (90-92) |  |
| Sex (Male/Female) | 67/53 | 41/31 | 11/6 | 0.806 |
| Smoking history (Present/Absent) | 63/56 | 40/31 | 12/4 | 0.249 |
| %VC <80%(Yes / No) | 19/92 | 10/58 | 4/12 | 0.590 |
| FEV1.0％<70% (Yes / No) | 41/67 | 26/36 | 5/9 | 0.840 |
| PNI (≥47 / <47) | 59/35 | 38/26 | 4/9 | 0.092 |
| Sarcopenia (Present / Absent) | 48/32 | 34/17 | 10/3 | 0.475 |
| SCS (≥9 / <9) | 31/89 | 26/45 | 8/9 | 0.936 |
| Histology (Adenocarcinoma / Sq / Others) | 83/32/5 | 43/23/6 | 12/5/0 | 0.543 |
| Clinical stage (I / II–IV) | 92/28 | 58/14 | 17/0 | 0.054 |
| Surgical approach (VATS / Open) | 90/18 | 62/8 | 14/2 | 0.636 |
| Surgical Procedure (Lobectomy/Segmentectomy/wedge resection) | 78/17/24 | 38/15/19 | 8/5/4 | 0.262 |
| Mediastinal LN dissection (Yes / No) | 68/43 | 35/31 | 9/7 | 0.562 |
| Values are presented as mean ± standard deviation or number of patients (%). IQR: Interquartile range; PNI: Prognostic Nutritional Index; SCS: Simplified Comorbidity Score; Sq: Squamous cell carcinoma; VATS: Video Assisted Thoracic Surgery. | | | | |

**Supplemental Table 2. Comparison between male and female in clinicopathological characteristics of the patients.**

| **Variables** | **Male** | **Female** | **p-value** |
| --- | --- | --- | --- |
| Total patients | 119 | 90 |  |
| Median age (IQR, years) | 86 (85-88) | 86 (85-88) | 0.512 |
| Smoking history (Present/Absent) | 107/10 | 8/81 | < 0.001 |
| %VC <80%(Yes / No) | 20/91 | 13/71 | 0.702 |
| FEV1.0％<70% (Yes / No) | 54/53 | 18/59 | < 0.001 |
| PNI (≥47 / <47) | 55/39 | 46/31 | 0.877 |
| Sarcopenia (Present / Absent) | 64/15 | 28/37 | < 0.001 |
| SCS (≥9 / <9) | 61/57 | 4/86 | < 0.001 |
| Histology (Adenocarcinoma / Sq / Others) | 61/51/7 | 77/9/4 | < 0.001 |
| Clinical stage (I / II–IV) | 92/27 | 75/15 | 0.301 |
| Surgical approach (VATS / Open) | 91/19 | 75/9 | 0.222 |
| Surgical Procedure (Lobectomy/Segmentectomy/wedge resection) | 68/19/32 | 56/18/15 | 0.210 |
| Mediastinal LN dissection (Yes / No) | 62/49 | 50/32 | 0.555 |
| Pathological stage (I / II–IV) | 88/31 | 70/20 | 0.626 |
| Postoperative complications (Yes / No) | 36/83 | 17/73 | 0.077 |
| Values are presented as mean ± standard deviation or number of patients (%). IQR: Interquartile Range; PNI: Prognostic Nutritional Index; SCS: Simplified Comorbidity Score; Sq: Squamous cell carcinoma; VATS: Video Assisted Thoracic Surgery. | | | |
